# Self-generating autocatalytic networks: structural results, algorithms and their relevance to early biochemistry

**DOI:** 10.1098/rsif.2023.0732

**Published:** 2024-05-22

**Authors:** Daniel Huson, Joana C. Xavier, Mike Steel

**Affiliations:** ^1^ Institute for Bioinformatics and Medical Informatics, Tübingen University, Tübingen, Germany; ^2^ Department of Chemistry, Imperial College London, London, UK; ^3^ Biomathematics Research Centre, University of Canterbury, Christchurch, New Zealand

**Keywords:** autocatalytic network, RAFs, algorithms, metabolic network

## Abstract

The concept of an autocatalytic network of reactions that can form and persist, starting from just an available food source, has been formalized by the notion of a reflexively autocatalytic and food-generated (RAF) set. The theory and algorithmic results concerning RAFs have been applied to a range of settings, from metabolic questions arising at the origin of life, to ecological networks, and cognitive models in cultural evolution. In this article, we present new structural and algorithmic results concerning RAF sets, by studying more complex modes of catalysis that allow certain reactions to require multiple catalysts (or to not require catalysis at all), and discuss the differing ways catalysis has been viewed in the literature. We also focus on the structure and analysis of minimal RAFs and derive structural results and polynomial-time algorithms. We then apply these new methods to a large metabolic network to gain insights into possible biochemical scenarios near the origin of life.

## 1. Introduction

A central property of the chemistry of living systems is that they combine two basic features: (i) the ability to survive on an ambient food source and (ii) each biochemical reaction in the system requires only reactants and a catalyst that are provided by other reactions in the system (or are present in the food set). The notion of a self-sustaining ‘collectively autocatalytic set’ captures these basic features, and their study was pioneered by Stuart Kauffman [1,2]. By investigating a simple binary polymer model, Kauffman showed that collectively autocatalytic sets invariably emerge once the network of polymers becomes sufficiently large.

The notion of a collectively autocatalytic set was subsequently formalized more precisely as a ‘reflexively autocatalytic and food-generated’ (RAF) set (defined shortly). RAF sets (RAFs) are related to, but somewhat different from Robert Rosen’s (M, R) systems (a partial connection between the two was described in [3]). RAF theory can also be investigated within the framework of Chemical Organization Theory (COT) [4]; for example, certain types of RAFs correspond to chemical organizations, as described in [5] (refer also to [6], §4).

RAF algorithms have also been used in the analysis of simple autocatalytic networks of polymers in laboratory studies, either from RNA molecules [7] or from peptides [8] and have been discussed further in modelling the origin of life (e.g. [9,10]). More recently, RAFs have also played a pivotal role in modelling self-reproduction and self-organization before the emergence of a genetic code in the polymer world. When modelling this early stage of chemical evolution [11,12], RAFs provide a framework for studying the organization of small molecules in complex chemical networks that would lead to, and support the growth of polymers as RNA and protein. A general framework to model complex catalysis is required here: some reactions can occur spontaneously (e.g. isomerizations), whereas some reactions may require multiple small molecules involved in catalysis. An example is the reaction catalysed by l-threonine dehydrogenase, important in amino acid metabolism, which requires an organic cofactor namely NAD, and a metal [13]. Moreover, in the data set analysed in [11], 1052 of the 5994 biochemical reactions involved catalyst combinations of two or more molecules to be present.

Two important features of the RAF approach are the degree of generality RAFs allow, and the fact that very large systems can be analysed precisely by fast algorithms. The generality of RAF theory means that a ‘reaction’ need not refer specifically to a chemical reaction, but to any process in which certain items are combined and transformed into new items, and where similar items facilitate (or catalyse) the process without being used up in the process. This has led to the application of RAF theory to processes beyond biochemistry, such as cognitive modelling in cultural evolution [14,15], ecology [16,17] and economics [17]. This generality is not unique to RAFs; for example, COT has been applied to diverse settings including sociology [18], ecology [19], cybernetics [20] and modelling of worldviews [21]. Petri nets have also been applied to biochemical modelling, including self-reproduction [22] and to other non-biochemical settings (e.g. [23]). In addition, a number of other recent structural approaches to autocatalytic networks have been applied in origins of life research [24,25].

In this article, we describe further extensions and applications of RAF theory. We describe an extension of the RAF approach that provides a unified handling of complex catalysis, leading to new mathematical results (§§2.2 and 3). We then focus on the structure and algorithmic properties of minimal RAFs in §4.1. Finally, in §5, we apply our theoretical results to investigate a large metabolic data set, thereby identifying new properties relevant to the emergence of early biochemistry.

## 2. Catalytic reaction systems

### 2.1. Reaction systems

A reaction system is a pair (X,R) consisting of a finite non-empty set X of elements (e.g. molecule types) and a finite set R of reactions. Here, a reaction r∈R refers to an ordered pair (A,B) where A and B are multisets of elements from X . We will write $r:a_{1}+\cdots +a_{k}\to b_{1}+\cdots +b_{l}$ to denote the reaction that has reactants $\left\{a_{1},\ldots ,a_{k}\right\}$ and products $\left\{b_{1},\ldots ,b_{l}\right\}$ . In applications, a bidirectional reaction is generally regarded as a pair of reactions (forward and backward) with the same catalysis assignment. We let ρ(r) denote the set corresponding to A (i.e. ignoring multiplicities) and π(r) denote the set corresponding to B (ignoring multiplicities); it is assumed implicitly that ρ(r),π(r)≠∅ . For a subset $R^{\prime }$ of R , it is convenient to let $\pi \left(R^{\prime }\right)={⋃}_{r\in R^{\prime }}\pi (r)$ denote the set of the products of the reactions in $R^{\prime }$ .

Next, consider a reaction system (X,R) together with a particular subset F of X . The set F can be interpreted as a set of elements that are freely available to the system; accordingly, F is referred to as a *food set*. A subset $R^{\prime }$ is *F-generated* if the reactions in $R^{\prime }$ can be placed in some linear order, say $r_{1},r_{2},\ldots ,r_{k}$ , such that the following property holds: for $\rho (r_{1})\subseteq F$ and for all values of j between 2 and k , we have $\rho (r_{j})\subseteq F\cup \pi (\{r_{1},\ldots ,r_{j-1}\})$ . In other words, the reactions in $R^{\prime }$ are F-generated if they can proceed in some order so that the reactant(s) of each reaction are available by the time they are first required. We call such an ordered sequence of $R^{\prime }$ an *admissible ordering*. Since there are k! ways to order k reactions, it may not be immediately obvious that the F-generated condition can be verified in polynomial time; however, there is a simple way to do so, as we now describe.

We first recall some further terminology. Given a subset $R^{\prime }$ of reactions R , a subset W of X is said to be $R^{\prime }$ *-closed* precisely when each reaction $r\in R^{\prime }$ that has all its reactant(s) in W also has all its the product(s) in W (i.e. $r\in R^{\prime },\rho (r)\subseteq W\Rightarrow \pi (r)\subseteq W$ ). The union of two closed sets need not be closed; nevertheless, given any non-empty subset *W*_0_ of X there is a unique minimal $R^{\prime }$ -closed set containing *W*_0_, denoted ${cl}_{R^{\prime }}(W_{0})$ . This can be computed in polynomial time in the size of the system by constructing a nested increasing sequence of subsets of the elements $W_{0}\subset W_{1},\ldots \subset W_{k}$ where:


$$W_{i+1}=W_{i}\cup \{x\in X:\exists r\in R^{\prime }:\rho (r)\subseteq W_{i},x\in \pi (r)\},$$

for i≥0 , and by letting $W_{k}$ denote the terminal set in this sequence (i.e. k is the first value of i for which $W_{i}=W_{i+1}$ ).

### 2.2. Catalytic reaction systems (allowing complex catalysis)

A catalytic reaction system (CRS) is a reaction system with a food set (X,R,F) together with a subset χ of $2^{X}\times R$ . Thus, χ is a collection of pairs (U,r) where U⊆X and r∈R . For (U,r)∈χ , we refer to U as a catalyst set for r . This is a generalization of earlier treatments in which χ consisted of a subset of X×R (i.e. simple catalysis by single elements). Our extension here to this more general catalysis framework allows for complex (i.e. conjunctive) catalysis rules, where catalysts of a reaction may require the presence of two or more elements of X (e.g. cofactors of enzymes). The treatment of complex catalysis in [26] required the introduction of fictitious new reactions and elements to the original CRS. Here, our more direct approach allows both simple and complex catalysis rules that require no additional reactions or elements to be introduced. It also permits the further option that particular uncatalysed reactions can appear in an autocatalytic system (since the definition of χ allows (∅,r)∈χ ), thereby addressing a recent concern discussed in §2.5.

We denote a CRS by writing 𝒬=(X,R,χ,F) , and we let |𝒬|=|X|+|R|+|χ| denote the *size* of 𝒬 , and we write


$$r:a_{1}+\cdots +a_{k}\,\left[U_{1},\ldots ,U_{m}\right]\to b_{1}+\cdots +b_{l}$$

to denote the reaction that has reactants $\left\{a_{1},\ldots ,a_{k}\right\}$ , products $\left\{b_{1},\ldots ,b_{l}\right\}$ and catalyst sets $\left\{U_{1},\ldots ,U_{m}\right\}$ , where $\left(U_{i},r\right)\in \chi$ for all i . When $U_{i}$ is a singleton set (say $\left\{c_{i}\right\}$ ), we will often write *c*_*i*_ in place of $\left\{c_{i}\right\}$ in our example systems.

### 2.3. RAFs

Given a CRS 𝒬=(X,R,χ,F) , a subset $R^{\prime }$ of R is said to be an *RAF* if $R^{\prime }$ is non-empty and if for each $r\in R^{\prime }$ , the reactants of r and at least one catalyst set U for r (as specified by χ ) is a subset of ${\mathrm{cl}}_{R^{\prime }}\,\left(F\right)$ .

An equivalent definition for a non-empty set $R^{\prime }$ to be an RAF for 𝒬 is that $R^{\prime }$ is F-generated, and each reaction $r\in R^{\prime }$ has a catalyst set U that is a subset of $F\cup \pi (R^{\prime })$ . A further equivalent definition is the following:

—
$R^{\prime }$ can be ordered $r_{1},r_{2},\ldots ,r_{k}$ so that for each i≥1 , the reactants of *r*_*i*_ are present in $X_{i}$ , where $X_{1}=F$ and $X_{i}=F\cup \pi (\{r_{1},\ldots ,r_{i-1}\})$ , and at least one catalyst set U of *r*_*i*_ is a subset of $X_{k}$ .

If a CRS 𝒬 has an RAF, then it has a unique maximal RAF (which is the union of all the RAFs for 𝒬 ), which is denoted maxRAF⁢(𝒬) . An RAF $R^{\prime }$ for 𝒬 is said to be an *irreducible* RAF (more briefly an *iRAF*) if $R^{\prime }\setminus \{r\}$ is not an RAF for 𝒬 (and contains no RAF for 𝒬 ) for each reaction *r* in $R^{\prime }$ .

A stronger notion than an RAF is a constructively autocatalytic and F-generated (CAF) set where the third equivalent definition of an RAF (above) is strengthened to ‘and at least catalyst set U of *r*_*i*_ is a subset of $X_{i}$ ’ (rather than ‘of $X_{k}$ ’). In other words, $R^{\prime }$ is a CAF if it has an admissible ordering in which at least one catalyst set has each of its elements already present in the food set or produced by an earlier reaction in the ordering. Every CAF is also an RAF, but the converse containment does not hold. Although RAFs and CAFs appear to be very similar concepts, they exhibit quite different properties. For example, if a CRS 𝒬 has a CAF, then this CAF must contain a reaction r for which all the reactants of r and at least one catalyst of r lie in F , in which case {r} is itself a CAF of size 1. By contrast, a large RAF need not contain any ‘small’ RAF within it. Moreover, theoretical and simulation studies on polymer systems reveal that the level of catalysis required for a CAF to be present is exponentially higher than that required for an RAF [27], and in real biochemical systems that have been studied (e.g. [11,28]), the maxRAF is generally not a CAF. Thus, in this article, we focus on the more general notion of an RAF.

### 2.4. Examples

We now describe three examples to illustrate the concepts above. The first example (from [7]) illustrates the concept of an RAF in the simpler setting where catalysis involves only singleton elements, the second example illustrates complex catalysis and the third example is from an experimental system.

**Figure 1.**
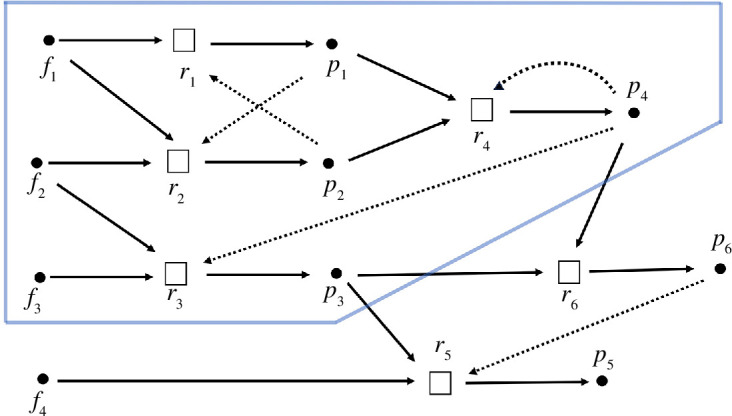
A simple example of a CRS with food set $\left\{f_{1},f_{2},f_{3},f_{4}\right\}$ six reactions (*r*_1_–r_6_) and with catalysis indicated via dashed arrows (adapted from [7]). The maxRAF consists of the four reactions within the blue border.

Consider the following CRS where $F=\left\{f_{1},f_{2},f_{3},f_{4}\right\}$ , $X=F\cup \left\{p_{1},\ldots ,p_{6}\right\}$ , and $R^{\prime }=\left\{r_{1},\ldots ,r_{6}\right\}$ indicated by squares in figure 1. In this figure, reactants and product pathways are indicated by solids arrows, and catalysis is indicated by dashed arrows. The maxRAF for this system consists of the four reactions $(r_{1}$ –*r*_4_), and there is one iRAF for this CRS, namely, $\left\{r_{1},r_{2}\right\}$ .

Next, consider the following CRS where X={a,b,c,d,e,g} , F={a,b} and $R=\left\{r_{1},r_{2},r_{3},r_{4}\right\}$ and χ are as follows:


$$r_{1}:a+a\;[\{c,d\},e]\to c,\;\;\;r_{2}:b+c\;[\emptyset ]\to d,\;\;\;\;\;r_{3}:b+b\;[\;]\to e,\;\;r_{4}:a+e\;[a]\to b,\;\;\;\;\;\;\;\;\;\;\;\;\;\;r_{5}:c+d\;[d]\to g+g.$$

Note the subtle difference between the catalyst set in *r*_2_ and *r*_3_. In *r*_2_ , we have χ={∅} , while in *r*_3_ , we have χ=∅ . This distinction is a way to formally allow certain reactions in an RAF to be uncatalysed if they proceed at a high rate without an additional element in the RAF acting as a catalyst (when χ={∅} ) and reactions that do not proceed fast enough without an additional element from the system acting as a catalyst (when χ=∅ ). This system has $\left\{r_{1},r_{2},r_{5}\right\}$ as its maxRAF, and $\left\{r_{1},r_{2}\right\}$ as its unique iRAF. No CAF is present in this CRS.

**Figure 2.**
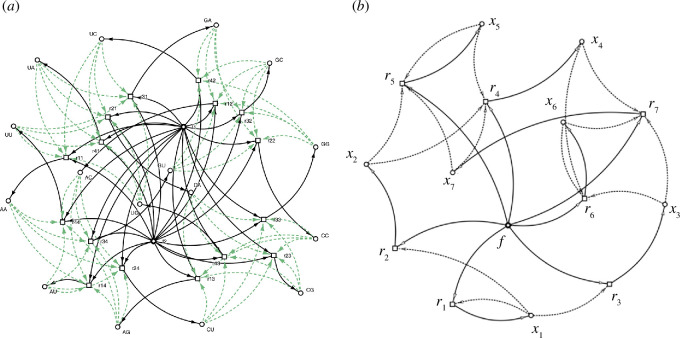
(*a*) An experimental RNA system described in [29] involving 16 reactions, and 18 molecule types (including a food set of size 2, which form the reactants of each reaction). The reactions are denoted by squares, elements (in this case, RNA replicators) are denoted by circles, reaction pathways are indicated by solid arrows, and catalysis is indicated by dotted arrows. This system forms an RAF [7]. (*b*) A subset of seven of the reactions from the full set (with the two food elements combined into a single element f ), which forms an RAF. This RAF analysed in [7] contains within it 67 other subsets that form RAFs including four irreducible RAFs (iRAFs). This RAF is not a CAF (nor does it contain one). Figures produced by *CatReNet* [30]

A third example of an RAF arising in an experimental system (involving simple rather than complex catalysis) is provided in figure 2.

### 2.5. The interpretation of catalysis and autocatalysis in RAFs

Note that, if (∅,r)∈χ , then r does not require any element of X for its catalysis, and so RAFs under our more general definition can contain uncatalysed reactions in settings where this is appropriate. This is particularly relevant, as a number of papers (e.g. [31–34]) have pointed to the restrictive nature of RAFs in requiring that all reactions in the RAF must be catalysed. For example, the authors of [31] stated:

‘Here, the assumption that all reactions are catalysed appears very unrealistic. Unfortunately, the algorithms for recognizing RAFs […] do not seem to generalize to arbitrary networks composed of non-catalysed reactions’.

and in [32], the author states:

‘The requirement that every reaction be catalyzed by another molecule seems too strong when we are dealing with simple metabolisms in the earliest forms of life. In modern organisms, almost every reaction of small-molecule metabolites is catalyzed by enzymes. However, prior to the existence of RNA and proteins, there were no enzymes, so we need a theory that deals with the reactions of small molecules without insisting that the reactions be catalyzed’.

In fact, in previous applications of RAF theory to the origins of metabolism [11,12] a fictional catalyst ‘Spontaneous’ was assigned to reactions known to occur uncatalysed. This catalyst was added to the food set in all simulations. Also, prior to the advent of genetic coding and enzymes, catalysis must have existed in the small-molecule world as well. Small molecules are increasingly being shown to catalyse multiple reactions in the absence of enzymes [35,36]. However, the use of a fictional catalyst ‘Spontaneous’ is less direct than our approach introduced here (see §2.2) where we generalize the notion of catalysis by describing it via a subset χ of $2^{X}\times R$ (thereby allowing pairs of the form (∅,r) , and so a ‘Spontaneous’ catalyst is no longer required).

RAF theory also treats ‘catalysis’ in a general way and this allows for efficient graph-theoretic algorithms, which apply independently of any detailed kinetic (or even stoichiometric) considerations. This generality also allows for applications in a variety of areas outside chemistry. Essentially, we regard a catalyst as any element that facilitates, speeds up or synchronizes a reaction without taking part in the reaction itself (as a reactant).

For example, in economic applications, a factory facilitates (i.e. catalyses) the production of items from the incoming raw materials, but is not itself consumed by that process. In cognitive modelling [14], a reaction that combines ideas to form a new idea could be enhanced (i.e. catalysed) by a need, thought, memory or stimuli. In ecology [16], a catalyst is a species that enables some other interaction in an ecological network. In biochemistry, it can also be helpful to treat catalysis in a quite general way; for instance, the formation of a lipid boundary to encompass a primitive metabolism can be viewed as a sequence of reactions which, once complete, forms a structural element (a complete lipid membrane) that catalyses all the reactions within the newly formed protocell (since the system within it no longer disperses) [37].

Moreover, in biochemistry, food elements can be direct catalysts for multiple reactions, as is the case with universally essential metal ions. The generality of RAF theory allows for this, in contrast to some other models of network autocatalysis as discussed in [38].

It is tempting to treat a catalyst x of a reaction r as simply an additional reactant and product of r (i.e. adding x to both sides of the reaction, as is sometimes used in chemical notation), thereby reducing the notion of an RAF to simply an F-generated subset of the resulting (modified) reaction system. In other words, the catalysed reaction x+y⁢[c]→z (where c is a catalyst) might be viewed as x+y+c→z+c . However, this misses an important distinction. Namely, in RAF theory, it is assumed that a reaction r may proceed (at a slow rate intially) provided that all its reactants are available even if no catalyst is initially available; however, r will subsequently be part of an RAF set, provided that a catalyst of r is (eventually) produced by at least one other reaction in the RAF. Indeed, it is entirely possible for a CRS to contain an RAF yet the modified reaction system (adding catalysts to both sides of a reaction) might have no F-generated subset (indeed, this has been observed in real biochemical networks [7,11,28]). Misunderstandings concerning the related notion of ‘autocatalysis’ have been discussed in [39] and [40].

## 3. Mathematical aspects of RAFs

We now recall the concept of the maxRAF operator φ (from [41], §3). For any subset $R^{\prime }$ of R , let $𝒬|R^{\prime }$ be the CRS $\left(X,R^{\prime },\chi^{\prime },F\right)$ where $\chi^{\prime }$ is the restriction of χ to $2^{X}\times R^{\prime }$ , and let $\varphi :2^{R}\to 2^{R}$ be the function defined as follows:


(3.1) $$\varphi (R^{\prime })=\left\{ \begin{array}{ll} maxRAF(Q|R^{\prime }), & \text{if }Q|R^{\prime }\text{ has a RAF};\\ \emptyset , & \text{otherwise}. \end{array}\right.$$

The function $\varphi \,\left(R^{\prime }\right)$ can be determined in a computationally efficient way, as follows:


$$\varphi (R^{\prime })=\underset{i\geq 1}{⋂}R_{i},$$

where $R_{1}=R^{\prime }$ and for i>1 ,


(3.2) $$R_{i}=\{r\in R_{i-1}:\rho (r)\subseteq {cl}_{R_{i-1}}(F)\text{ and }(U,r)\in \chi \;\text{ for some }U\subseteq {cl}_{R_{i-1}}(F)\}.$$

Notice that the sets $R_{i}$ defined in equation (3.2) form a decreasing nested sequence, and so $\varphi \,\left(R^{\prime }\right)$ is precisely the set $R_{j}$ for the first value of j≥1 for which $R_{j+1}=R_{j}$ . In particular, a non-empty subset $R^{\prime }$ of R is an RAF if and only if $\varphi \,\left(R^{\prime }\right)=R^{\prime }$ . Moreover, φ⁢(R) is precisely the maxRAF of 𝒬 (if it exists), or is the empty set otherwise.

**Example 3.1.** To illustrate this algorithm, consider the second example described in §2.4. For $R^{\prime }=R=\left\{r_{1},\ldots ,r_{5}\right\}$ , we have $R_{1}=R$ , and so ${\mathrm{cl}}_{R_{1}}\,\left(F\right)=\left\{a,b,c,d,e,g\right\}$ , and all but one of the reactions (namely, *r*_3_) satisfy the property that $\left(U,r)\in \chi \;\text{ for some }U\subseteq {cl}_{R_{1}}(F\right)$ . Thus by equation (3.2), $R_{2}=\left\{r_{1},r_{2},r_{4},r_{5}\right\}$ . Next, $R_{3}=\left\{r_{1},r_{2},r_{5}\right\}$ since one of the reactants of $r_{4}\in R_{2}$ (namely, e ) is not present in ${\mathrm{cl}}_{R_{2}}\,\left(F\right)$ . Finally, $R_{4}=R_{3}$ , and so $\varphi \,\left(R\right)=R_{3}$ forms the maxRAF for 𝒬 .

A number of interesting algebraic (semigroup) properties of the map φ have been explored recently in [42] (also refer [43], which considers a more general notion than an RAF, which corresponds to what have been called ‘pseudo-RAFs’ in the RAF literature).

The map φ is also an example of the more general notion of an interior operator (on $2^{R}$ ), and several basic results in RAF theory can be derived from this property alone. To explain this further, an arbitrary function $\psi :2^{Y}\to 2^{Y}$ is said to be an *interior operator^1^* if it satisfies the following three properties (nesting, monotonicity and idempotence) for all subsets $A,A^{\prime }$ of Y :

— (*I*_1_): ψ⁢(A)⊆A ,
— (*I*_2_): $A\subseteq A^{\prime }\Rightarrow \psi \,\left(A\right)\subseteq \psi \,\left(A^{\prime }\right)$ and
— (*I*_3_): ψ⁢(ψ⁢(A))=ψ⁢(A) .

**Lemma 3.1.** *Given any CRS* 𝒬=(X,R,χ,F) *, the maxRAF function* $\varphi :2^{R}\to 2^{R}$ *is an interior operator.*

Many results of RAF theory (including some we discuss later) depend mainly on the interior operator property of φ . A natural question is whether *any* interior operator on a finite set can be represented as the maxRAF operator of a set of reactions associated with the elements of the set. It was recently shown that if Y is a finite set of size at least 12, then there exists an interior operator on Y that cannot be represented as a maxRAF operator of a suitably chosen CRS $\left(X,R_{Y},\chi ,F\right)$ where $R_{Y}$ is a set of reactions bijectively associated with Y and where $\chi \subseteq X\times R_{Y}$ (i.e. the original CRS setting which does not allow complex catalysis rules) [44]. Thus, some generic properties of RAFs in this setting cannot be established by using the interior operator properties alone. However, if one allows complex catalysis rules, we have the following contrasting result (further details and proof are provided in appendix A).

**Proposition 3.1.** *For any finite set* Y *, any interior operator* ψ *on* $2^{Y}$ *can be represented as the maxRAF operator* φ *for a suitably chosen CRS* $𝒬=\left(X,R_{Y},\chi ,F\right)$ *, where* $R_{Y}$ *is bijectively associated with* Y *and* $\chi \subseteq 2^{X}\times R_{Y}$ *permits complex catalysis rules. Moreover,* $R_{Y}$ *can be chosen so that each of its reactions has the same single reactant* f *(from the food set) and a single associated product.*

Proposition 3.1 has an interesting consequence for the structure of RAFs in any CRS. Specifically, it implies that it is possible to simplify the reactions and the food set, while preserving the number, sizes and containment structure of the RAFs (albeit at the price of possibly increasing substantially the number and complexity of catalysts for the reactions). More precisely, we have the following result (obtained from proposition 1 by taking ψ to be the maxRAF operator of 𝒬 ).

**Corollary 3.1*.*** *For any CRS* 𝒬=(X,R,χ,F) *, there is a matching CRS* ${𝒬}^{\prime }=\left(X^{\prime },R^{\prime },\chi^{\prime },F^{\prime }\right)$ *, where (i) each of the reactions in* $R^{\prime }$ *has a single reactant* f *which comprises the food set* F *, and a single distinct product and (ii) there is a canonical bijective correspondence between* R *and* $R^{\prime }$ *that induces a bijection between the RAFs of* 𝒬 *and the RAFs of* ${𝒬}^{\prime }$ *.*

A worked example to illustrate this result is provided in appendix A. We return to interior operators in §4.2.

### 3.1. Strictly autocatalytic RAFs

If a CRS 𝒬 has the property that each reaction is catalysed by at least one element of the food set, then the RAF sets for 𝒬 coincide precisely with the F-generated sets. More generally, for any CRS, it is possible for an RAF set to have the property that some (or all) of its reactions are catalysed by elements of the food set, which renders the notion of ‘autocatalytic’ less applicable (though not entirely, since each reaction in the RAF might still also be catalysed by at least one product of the other reactions in the RAF). To formalize this notion further, we introduce a new definition and describe a result that characterizes the condition which captures a more focused notion of an ‘autocatalytic’ RAF.

Given a CRS 𝒬=(X,R,χ,F) , we say that a subset $R^{\prime }$ of R is *strictly autocatalytic* if each reaction in $R^{\prime }$ has at least one catalyst that involves one or more products of reactions of $R^{\prime }$ and is not a subset of the food set. We say $R^{\prime }$ is a strictly autocatalytic RAF for 𝒬 if $R^{\prime }$ is an RAF and is strictly autocatalytic.

**Example 3.2.** Consider the following catalytic reaction system:


$$r_{1}:a+b\;[\{a,d\}]\to e\;\;\;\;\;\;\;r_{2}:b+c\;[\{a,b\},e]\to d\;\;\;\;\;r_{3}:d\;[\{a,b\}]\to c\;\;\;\;\;\;\;\;\;\;\;$$

where X={a,b,c,d,e} and F={a,b,c} . Then, $\left\{r_{2}\right\},\left\{r_{1},r_{2}\right\},\left\{r_{2},r_{3}\right\}$ and $\left\{r_{1},r_{2},r_{3}\right\}$ are RAFs, however $\left\{r_{1},r_{2}\right\}$ is the only strictly autocatalytic RAF.

Let ${\hat{R}}_{i},i\geq 1$ be the nested decreasing sequence of subsets of R defined as follows: ${\hat{R}}_{1}=R$ , and for i>1 , consider the following modification of equation (3.2):


(3.3) $${\hat{R}}_{i}=\{r\in {\hat{R}}_{i-1}:\rho (r)\subseteq {cl}_{{\hat{R}}_{i-1}}(F)\text{ and }(U,r)\in \chi \;\text{ for some }U\subseteq {cl}_{{\hat{R}}_{i-1}}(F)\text{ and }U⊈F\}.$$

The following result shows that there is a polynomial-time algorithm (in |𝒬| ) to determine whether or not 𝒬 has a strictly autocatalytic RAF and, if so, to construct a maximal one. The proof is provided in appendix A.

**Proposition 3.2*.*** *Let* 𝒬=(X,R,χ,F) *be a CRS. Then* 𝒬 *has a strictly autocatalytic RAF if and only if* ${⋂}_{i\geq 1}\hat{R_{i}}\neq \emptyset$ *, in which case,* ${⋂}_{i\geq 1}\hat{R_{i}}$ *is the unique maximal strictly autocatalytic RAF for* Q *.*

## 4. Irreducible (minimal) RAFs

Recall that an iRAF is an RAF that contains no RAF as a proper subset. Such RAFs are of particular interest, as they represent the minimal possible autocatalytic networks within a CRS. The smallest-sized RAF in any CRS is necessarily an iRAF, however, iRAFs can be of different sizes. Moreover, even without complex catalysis rules, a CRS 𝒬 can have exponentially many iRAFs, and although finding one iRAF is easy, finding a smallest one turns out to be NP-hard [6].

### 4.1. Identifying all iRAFs in a CRS

Let 𝒬=(X,R,χ,F) be a fixed CRS that contains an RAF. Recall that an RAF $R^{\prime }$ for 𝒬 is said to be an iRAF if $R^{\prime }\setminus \{r\}$ is not an RAF for 𝒬 (and contains no RAF for 𝒬 ) for each reaction $r\in R^{\prime }$ . Every RAF of a CRS contains an iRAF, and there is a simple (polynomial-time in |𝒬| ) algorithm for finding one or more iRAFs within any given RAF; however, the number of iRAFs for 𝒬 can grow exponentially with |𝒬| (theorem 1 of [45]). Moreover, determining the size of a *smallest* iRAF is known to be NP-hard [6]. Nevertheless, there is a simple (and polynomial-time) algorithm to determine whether 𝒬 has just one iRAF; we simply ask whether the set


Core⁢(𝒬):={r∈R:φ⁢(R∖{r})=∅}

is an RAF. Then it can be shown that this set is an RAF if and only if 𝒬 has a unique iRAF, and in that case, C⁢o⁢r⁢e⁢(𝒬) is the unique iRAF for 𝒬 . The result for CRS systems without complex catalysis was established in [41, theorem 4.1], however, since the proof involved only interior operator properties for φ it applies to the more general setting here (by lemma 3.1).

Moreover, it can easily be seen that there is a simple way to test whether or not an iRAF *R*_1_ is the only iRAF for 𝒬 ; this holds provided that:


$$\varphi (\varphi (R)\setminus \{r\})=\emptyset ,\text{ for all }r\in R_{1}.$$

This is computationally less-intensive than computing C⁢o⁢r⁢e⁢(𝒬) , since it involves searching over the reactions that are just in *R*_1_ rather than all of R .

In a similar way, it can be shown that if 𝒬 has (at least) two iRAFs, *R*_1_ and *R*_2_ then these are the only iRAFs for 𝒬 if and only if for all $r\in R_{1}\setminus R_{2}$ , we have $\varphi \,\left(\varphi \,\left(R\right)\setminus \left\{r\right\}\right)=R_{2}$ , and for all $r\in R_{2}\setminus R_{1}$ , we have $\varphi \,\left(\varphi \,\left(R\right)\setminus \left\{r\right\}\right)=R_{1}$ .

A direct extension of this last result to three or more iRAFs is problematic, since although two iRAFs cannot be nested (i.e. neither can be a subset of the other) in the case of three iRAFs, it is possible for each one to be a subset of the union of the two others. An example is provided by the CRS 𝒬=(X,R,χ,F) , where F={f} , $R=\left\{r_{1},r_{2},r_{3}\right\}$ and


$$r_{1}:f\,\,\left[x_{1}\right]\to x_{2}+x_{3}$$

$$r_{2}:f\,\,\left[x_{2}\right]\to x_{1}+x_{3}$$

$$r_{3}:f\,\,\left[x_{3}\right]\to x_{1}+x_{2}$$

This system has three iRAFs, namely, $\left\{r_{1},r_{2}\right\},\left\{r_{1},r_{3}\right\}$ and $\left\{r_{2},r_{3}\right\}$ , each of which is contained in the union of the other two.

However, the following result shows that testing a small number (say, k ) of iRAFs is feasible in polynomial time in |𝒬| (provided that k is fixed). The result is a slight strengthening of theorem 2 of [6]; its significance is that it allows one to determine whether a given set of k iRAFs is the set of *all* iRAFs for 𝒬 in polynomial time in |𝒬| , provided that k is fixed (however, the algorithm is exponential in k ). The proof of the following result is given in appendix.

**Proposition 4.1.** *A given collection* $R_{1},\ldots ,R_{k}$ *of iRAFs for* 𝒬 *is the set of all iRAFs for* 𝒬 *if and only if the following condition holds: for all choices* $\left(r_{1},\ldots ,r_{k}\right)$ *where* $r_{i}\in R_{i}$ *, we have* $\varphi \,\left(\varphi \,\left(R\right)\setminus \left\{r_{1},\ldots ,r_{k}\right\}\right)=\emptyset$ *.*

Proposition 4.1 assumes that the iRAFs are given in advance (e.g. found by heuristic search); however, the approach can be extended to identify the complete set of iRAFs of 𝒬 even if they are not given in advance.

Thus, when the number of iRAF is small (say, less than some value k ), the following algorithm is polynomial-time in |𝒬| (but is exponential in k ). The algorithm proceeds as follows and when it terminates, it produces the complete list of iRAFs of 𝒬 .

— Given 𝒬=(X,R,χ,F) , determine the maxRAF; providing that it exists, compute an iRAF, denoted *R*_1_.
— For i≥1 , construct a sequence of iRAFs starting with *R*_1_, as follows.
  - For eachinteger j with 1≤j≤i and each choice of i reactions $\left\{r_{1},r_{2},\ldots ,r_{i}\right\}$ where $r_{j}\in R_{j}$ for each j , compute $\varphi \,\left(\varphi \,\left(R\right)\setminus \left\{r_{1},\ldots ,r_{i}\right\}\right)$ . If this set is the empty set for all such choices of $\left\{r_{1},\ldots ,r_{i}\right\}$ , then $R_{1},\ldots ,R_{i}$ is the complete set of iRAFs for 𝒬 , so the algorithm terminates.
  - Otherwise, if $\varphi \,\left(\varphi \,\left(R\right)\setminus \left\{r_{1},\ldots ,r_{i}\right\}\right)\neq \emptyset$ for some choice of $\left\{r_{1},\ldots ,r_{i}\right\}$ , then compute an iRAF of $\varphi \,\left(\varphi \,\left(R\right)\setminus \left\{r_{1},\ldots ,r_{i}\right\}\right)$ and set $R_{i+1}$ equal to this iRAF, and proceed to step (iii).
  - Repeat step (i) and, if necessary, step (ii).

### 4.2. Finding an iRAF that contains a given reaction

Given any CRS 𝒬=(X,R,χ,F) , a natural question is whether a given reaction in R is present in at least one iRAF for 𝒬 . We show shortly that this problem is NP-hard. First, we describe how the union of all the iRAFs present in a CRS can be described via a further interior operator. Given a CRS 𝒬=(X,R,χ,F) , and a non-empty subset $R^{\prime }$ of R , let $\tilde{\varphi }\,\left(R^{\prime }\right)$ denote the union of the subsets of $R^{\prime }$ that are iRAFs for 𝒬 . The proof of the following lemma is given in appendix A, as a consequence of a more general result.

**Lemma 4.1*.*** *The map* $\tilde{\varphi }:2^{R}\to 2^{R}$ *is an interior operator.*

Although the computation of $\varphi \,\left(R^{\prime }\right)$ is polynomial time in |𝒬| for any CRS 𝒬=(X,R,χ,F) and any subset $R^{\prime }$ of R , computing $\tilde{\varphi }\,\left(R\right)$ is NP-complete, even for quite simple CRS systems, as we now state more formally (a proof is provided in the Appendix).

**Proposition 4.2*.*** *Given a CRS* 𝒬=(X,R,χ,F) , *the problem of determining whether or not a given reaction* r∈R *is present in at least one iRAF for* 𝒬 *is NP-complete. Moreover, this holds even for systems where each reaction in* R *has the same single food-set reactant and where each catalyst consists of single elements (i.e. without complex catalysis).*

### 4.3. Finding a minimal RAF that generates given elements

Next suppose we have a CRS 𝒬=(X,R,χ,F) and elements $x_{1},\ldots ,x_{k}$ in X∖F , each of which is produced by some reaction in maxRAF⁢(𝒬) . A relevant question in certain applications is to find a minimal subset $R^{\prime }$ of reactions within maxRAF⁢(𝒬) that is both an RAF and produces these particular elements of interest (e.g. molecule types that play a key role in a metabolic network, such as amino acids, the universal building blocks of proteins). In other words, $R^{\prime }$ is an RAF that produces $x_{1},\ldots ,x_{k}$ , and every proper subset of $R^{\prime }$ either fails to be an RAF or fails to produce all of the specified elements. It turns out that finding such a minimal set has a polynomial-time solution, as we now show (the proof is provided in appendix).

**Proposition 4.3*.*** *Let* ${𝒬}^{+\left\{x_{1},\ldots ,x_{k}\right\}}$ *be the CRS obtained from* 𝒬 *by replacing each catalyst* (U,r)∈χ *with* $\left(U\cup \left\{x_{1},\ldots ,x_{k}\right\},r\right)$ *. The collection of minimal subsets of* maxRAF⁢(𝒬) *that are simultaneously RAFs of* 𝒬 *and produce each of the elements* $x_{1},\ldots ,x_{k}$ *are precisely the iRAFs of* ${𝒬}^{+\left\{x_{1},\ldots ,x_{k}\right\}}$ *.*

### 4.4. Describing an RAF in terms of a composition sequence involving iRAFs

Any RAF contains the union of its iRAFs; however, it may be strictly larger than this union. A simple example is provided by the simple catalytic reaction system:


$$r_{1}:f\,\,\left[y\right]\to y$$

$$r_{2}:y\,\,\left[z\right]\to z$$

where F={f} and X={f,y,z} . This system is an RAF but its only iRAF is $\left\{r_{1}\right\}$ . In general, an RAF $R^{\prime }$ is the union of its iRAFs if and only if each reaction in $R^{\prime }$ is contained in an iRAF.

Nevertheless, any RAF $R^{\prime }$ in a CRS 𝒬=(X,R,χ,F) can be described by a sequence of iRAFs and associated catalytic reaction systems. This is loosely analogous to the description of finite groups in abstract algebra via a ‘composition series’, in which a group is reduced to the trivial group via quotients that are simple groups. Here, the analogue of a group (respectively, a simple group) is a subset of reactions (respectively, an iRAF), and the analogue of a quotient group is the complement of a iRAF in a set of reactions. However, this analogy is only suggestive; for a finite group, the set of associated simple groups is uniquely determined by the group, however, we do not expect the same uniqueness to hold concerning the set of associated iRAFs in our decomposition.

To describe this in our setting, suppose we have a CRS, 𝒬=(X,R,χ,F) and any non-empty subset $R^{\prime }\subseteq R$ . A *composition sequence* for $R^{\prime }$ is a nested decreasing sequence of subsets of $R^{\prime }$ , $R_{1},\ldots ,$ with $R_{1}=R^{\prime }$ and with $R_{i+1}=R_{i}\setminus {\hat{R}}_{i}$ where ${\hat{R}}_{i}$ is an iRAF of ${𝒬}_{i}=\left(X,R_{i},\chi ,F\cup \pi \,\left({\hat{R}}_{i-1}\right)\right)$ for each i≥1 for which ${𝒬}_{i}$ has a (non-empty) RAF. Since the sets $R_{i}$ form a nested decreasing sequence of sets, we refer to the final distinct set (i.e. ${⋂}_{i\geq 1}R_{i}$ ) as the *terminal set* of the sequence.

It is clear that every subset $R^{\prime }$ of R has a composition sequence, and that this can be constructed in polynomial time in |𝒬| . Moreover, by definition, the iRAF sets ${\hat{R}}_{i}$ are disjointed. In the simple two-reaction example above, the unique composition sequence for $R^{\prime }=\left\{r_{1},r_{2}\right\}$ is: $\left\{r_{1},r_{2}\right\},\left\{r_{2}\right\},\emptyset$ . The proof of the following result is provided in appendix A.

**Proposition 4.4.** *Let* 𝒬=(X,R,χ,F) *be a CRS, let* $R^{\prime }$ *be a non-empty subset of* R *, and let* $R_{1},R_{2},\ldots$ *be a composition sequence for* $R^{\prime }$ *. The following are equivalent:*

-
$R^{\prime }$ *is an RAF for* 𝒬 *;*
-
$R_{1},R_{2},\ldots$ *has terminal set* ∅ *.*
- *The associated iRAF sets* $\left({\hat{R}}_{i},i\geq 1\right)$ *partition* $R^{\prime }$ *.*

Note that the iRAF sets ${\hat{R}}_{i}$ in (iii) are not, in general, iRAFs of the original CRS 𝒬 , and since the sets ${\hat{R}}_{i}$ are (by definition) disjoint, the partitioning condition in (iii) is equivalent to the condition that the union of the sets ${\hat{R}}_{i}$ is equal to $R^{\prime }$ .

## 5. Relevance to two questions in early biochemistry

As noted in §1, RAF theory is particularly interesting in the context of abiogenesis and early life, as it allows the direct investigation of autocatalysis in biochemical networks without the need for detailed kinetic knowledge, among other advantages [12]. To investigate the application of the new results proposed here to early biochemistry, we used the large metabolic dataset compiled in [11], constituting 6039 reactions. The food set used was the ‘rich’ food set of size 68 which in that paper resulted in a maxRAF of 1357 reactions.

The first question we addressed was: is there a biochemically relevant autocatalytic network that is strictly autocatalytic? This is where all the catalysts necessary for the network’s persistence are produced by the network. We implemented the strictly autocatalytic RAF algorithm described by proposition 3.2, which determined that no such strictly autocatalytic RAF exists in global metabolism, as described by this dataset. This, in turn, implies that in any RAF for this system, one or more elements in the food set play a pivotal role in catalysing some reaction(s). This is consistent with the aforementioned essentiality of metals in biocatalysis in all of metabolism [38]. At the same time, this result also reveals that there must exist unknown prebiotic routes (or unknown non-enzymatic catalysts) to the production of organic cofactors which need to be investigated in the laboratory. For example, there are no routes in this network to produce organic cofactors such as NAD [12] and the search for the prebiotic route for its synthesis is the subject of active investigation [46,47]. The dependence of life on its environment is deeply rooted in its geochemical origins, as demonstrated here by the structure of biochemical autocatalysis.

The second question concerns whether key elements important in biochemistry can be generated by small autocatalytic networks. This is often referred to as ‘minimal’ network exploration and is of particular interest for experimental researchers looking for routes to produce key prebiotic molecules in the laboratory. An important group of these key molecules is the set of 20 amino acids, which are the universal building blocks of proteins. A large subset of these had to exist in abundance prior to the origins of genetic coding. In [11], 17 out of the universal 20 amino acids could be produced by the maxRAF (which contained 1357 reactions). Here, we used proposition 4.5 to investigate the size of the minimal RAF that produces these 17 amino acids (both singly and together). In order to produce all 17 amino acids, the minRAF turns out to be of size 74, while for each amino acid separately, the minRAF sizes range from 1 to 28 and are shown in table 1. This and all individual minRAFs’ compositions are provided in electronic supplementary material, data 1.

**Table 1.** Seventeen amino acids that can be obtained via small-molecule autocatalysis using known biochemistry and respective minimal RAF size.

amino acid	minRAF size	amino acid	minRAF size
l-Methionine	1	l-Glutamate	7
l-Cysteine	1	l-Glutamine	8
l-Serine	2	l-Lysine	10
Glycine	3	l-Proline	11
l-Alanine	3	l-Leucine	12
l-Threonine	3	l-Isoleucine	15
l-Aspartate	4	l-Tyrosine	21
l-Asparagine	5	l-Tryptophan	28
l-Valine	7		

The results are consistent with existing biochemical knowledge and suggest routes for exploration of prebiotically plausible amino acid synthesis. The results also point to the limitations of current biochemical knowledge. One example of this limitation is the one-reaction obtained to produce l-methionine (Met), which derives it from *S*-adenosyl-methionine (SAM), a more complex cofactor present in the rich food set, making it an unreasonable prebiotic route. When SAM is removed from the food set, Met can still be produced, and the minRAF size expands to 12 reactions. In the case of cysteine, though, the reaction is, at first sight, prebiotically plausible. Simpler precursors (H_2_S, pyruvate and ammonia) are used in the reverse direction of a cysteine degradation reaction, to form cysteine and water. Even though all chemical reactions are theoretically reversible, it is quite unlikely that this one can occur in this direction exactly as shown, due to thermodynamic and mechanistic constraints. However, it is not impossible to envision a similar route via intermediates as phosphoenolpyruvate; computational chemistry may help by searching the space around the proposed route.

A more straightforward positive example is that of l-alanine. The minRAF found includes two reactions to produce reducing power (via NADH), plus the actual reaction that goes from pyruvate, ammonia, H^+^ and NADH to produce alanine. It has been known that pyruvate can lead to alanine in different experimental conditions [36], which this result hints at. Similarly, the minRAF obtained for l-aspartate points to the known short route to this amino acid via oxaloacetate [48]. Finally, it is interesting and consistent to observe that l-tryptophan and l-tyrosine have the largest minRAFs (21 and 28 reactions, respectively), as these aromatic amino acids are more complex and thought to appear later in prebiotic evolution. Note that, the complex amino acids histidine, phenylalanine and arginine cannot be produced with this dataset with small-molecule catalysis only. In summary, our results are a guide to explore the space around biochemistry and link it to plausible geochemical routes for the origins of life.

## 6. Concluding comments

Autocatalytic networks have provided a formal tool to investigate processes in early biochemistry and certain other settings that involve the formation and evolution of complex structures. The discrete nature of the model provides a way to develop and implement efficient algorithms that can be applied to large data sets, and to elucidate their structural properties, such as their building blocks in terms of iRAFs or the ordering of reactions.

In this article, we have developed further techniques that open the door to more detailed investigations into the structural properties of RAFs and iRAFs, their extensions to more general catalytic scenarios, and the investigation of the constraints on the order of reactions and the appearance of particular elements of interest. Several of the techniques described here have been implemented in the open-source software package *CatReNet* [30], which we plan to apply to investigate further aspects of early metabolism and related evolutionary questions.

## Data Availability

Data are available from the electronic supplementary material [50].

## Footnotes

^1^
The term `interior operator’ comes from topology, since the function that assigns to any subspace S of a topological space the topological interior of S satisfies the three properties described.

## Appendix A. Mathematical details

***Proof of lemma 3.1.*** For a subset $R^{\prime }$ of R , let

$$\lambda (R^{\prime })=\{r\in R^{\prime }:\rho (r)\subseteq {cl}_{R^{\prime }}(F)\text{ and }(U,r)\in \chi \text{ for some }U\subseteq {cl}_{R^{\prime }}(F)\}.$$

It is clear that the function λ satisfies properties (*I*_1_) and (*I*_2_) (but not necessarily (*I*_3_)), and since $\varphi \,\left(R^{\prime }\right)={⋂}_{i\geq 1}H_{i}\,\left(R^{\prime }\right)$ where $H_{1}\,\left(R^{\prime }\right)=R^{\prime }$ and $H_{i+1}\,\left(R^{\prime }\right)=\lambda \,\left(H_{i}\,\left(R^{\prime }\right)\right)$ for all i≥1 , property $\left(I_{3}\right)$ holds by proposition 1 of [44]. ∎

***Proof of proposition 3.1*** **.** Let ψ be an arbitrary interior operator on $2^{Y}$ . We construct a CRS ${𝒬}_{\psi }=\left(X,R_{Y},\chi ,F\right)$ that permits complex catalysis rules, as follows. Let F={f} , let $X=\left\{x_{y}:y\in Y\right\}\cup \left\{f\right\}$ , and let $R_{Y}=\left\{r_{y}:y\in Y\right\}$ where *r*_*y*_ is the reaction $f\to x_{y}$ . It remains to describe, for each reaction $r_{y}\in R_{Y}$ , its associated catalyst sets (i.e. the subsets U of X for which $\left(U,r_{y}\right)\in \chi$ ). Let

(A 1)
C⁢(y)={A⊆Y:y∈A,ψ⁢(A)=A},

the fixed sets of ψ that contain y . Then we let $\left(U,r_{y}\right)$ be an element of χ if and only if $U=\left\{x_{a}:a\in A\right\}$ for some set A∈C⁢(y) .

Let $b:Y\to R_{Y}$ denote the bijection $y\mapsto r_{y}$ .

**Claim 3.1.** A non-empty set A of Y is a fixed set of ψ if and only if $b\,\left(A\right)=\left\{r_{y}:y\in A\right\}$ is an RAF for ${𝒬}_{\psi }$ .

To establish this claim, suppose first that A is a fixed set of ψ (i.e. ψ⁢(A)=A ). Since any non-empty subset of $R_{Y}$ is F -generated (because each reaction in $R_{Y}$ has only the single reactant f which lies in the food set), it suffices to show that each reaction *r*_*y*_ in b⁢(A) has an associated catalyst set U with the property that each of its elements is produced by at least one reaction in b⁢(A) . Suppose that $r_{y}\in b\,\left(A\right)$ . Then y∈A , and, by assumption, A is a fixed set of ψ , so A∈C⁢(y) ; moreover, the elements $\left\{x_{a}:a\in A\right\}$ are products of reactions in b⁢(A) . Thus, taking $U=\left\{x_{a}:a\in A\right\}$ , we have $\left(U,r_{y}\right)\in \chi$ ; in other words, *r*_*y*_ is catalysed by products of reactions in b⁢(A) . Since this holds for all $r_{y}\in b\,\left(A\right)$ , it follows that b⁢(A) is an RAF.

Conversely, suppose that b⁢(W) is an RAF for ${𝒬}_{\psi }$ for some subset W of Y . Then for each w∈W , the reaction $r_{w}:f\to x_{w}$ has a catalyst set of the form $\left\{x_{a}:a\in A_{w}\right\}$ where $A_{w}$ is a non-empty subset of W satisfying $w\in A_{w}$ and $\psi \,\left(A_{w}\right)=A_{w}$ . It remains to show that ψ⁢(W)=W . Since ψ(W)⊆W it suffices to determine the converse containment. We have:

$$W\subseteq \underset{w\in W}{⋃}A_{w}=\underset{w\in W}{⋃}\psi \,\left(A_{w}\right)=\psi \,\left(\underset{w\in W}{⋃}A_{w}\right)\subseteq \psi \,\left(W\right),$$

as required to establish claim 3.1.

Next observe that the RAFs for ${𝒬}_{\psi }$ are simply the non-empty fixed sets of φ (the maxRAF operator on $R_{Y}$ for the CRS $Q_{\psi }$ ), and so ψ and $b^{-1}\circ \varphi \circ b$ are two interior operators on $2^{Y}$ , and these two functions the same fixed sets by claim 1 (noting also that φ and ψ also trivially fix ∅ ). However, any two interior operators on $2^{Y}$ that have the same collection of fixed sets are identical functions. This is because any interior map ν on a set $2^{Y}$ is completely determined by its fixed sets since for any set $Y^{\prime }\subseteq Y$ we have:

$$\nu \,\left(Y^{\prime }\right)=⋃\left\{U\subseteq Y^{\prime }:\nu \,\left(U\right)=U\right\}.$$

Thus $\psi =b^{-1}\circ \varphi \circ b$ , completing the proof. ∎

**Remark 3.1.** It can be shown that claim 3.1 also holds if we restrict the catalyst sets $U=\left\{x_{a}:a\in A\right\}$ to just those that correspond to the *minimal* sets A∈C⁢(y) (where minimal refers to set inclusion).

*An example to illustrate corollary 3.1:* Consider the following CRS (based on Kauffman’s binary polymer model with food set F={0,1,00,01,10,11} from fig. 1 of [26]):

$$r_{1}:10+0\;[01100]\to 100\;\;\;\;\;\;\;r_{2}:01+100\;[0]\to 01100\;\;\;\;\;\;\;r_{3}:10+1\;[0]\to 101\;\;\;\;\;\;\;\;\;\;\;\;\;r_{4}:11+10\;[101]\to 1110\;\;\;\;\;\;\;r_{5}:1110+0\;[101]\to 11100\;\;\;$$

This system is itself an RAF and it contains six other RAFs as subsets, namely $\left\{r_{1},r_{2}\right\},\left\{r_{3}\right\},\left\{r_{1},r_{2},r_{3}\right\},\left\{r_{3},r_{4}\right\},\left\{r_{3},r_{4},r_{5}\right\},\left\{r_{1},r_{2},r_{3},r_{4}\right\}$ . Following the procedure described in the proof, we associate with *r*_*i*_ the reaction $r_{i}^{\prime }:f\,\left[U_{i}\right]\to x_{i}$ where the set of (complex) catalysts is $U_{i}=\{\{x_{j}:r_{j}\in A\}:A\;\text{ is a RAF containing }\;r_{i}\}$ . For example, for *r*_1_ we have:

$$U_{1}=\left\{\left\{x_{1},x_{2}\right\},\left\{x_{1},x_{2},x_{3}\right\},\left\{x_{1},x_{2},x_{3},x_{4}\right\},\left\{x_{1},x_{2},x_{3},x_{4},x_{5}\right\}\right\}.$$

We can further simply *U*_1_ in line with the remark following the proof of proposition 1 above. For *U*_1_ we only need to keep the single set $\left\{x_{1},x_{2}\right\}$ since all other sets in *U*_1_ contain this set. Applying this simplification to the other sets $U_{i}$ leads to the resulting reduced system:

$$r_{1}^{\prime }:f\;[\{x_{1},x_{2}\}]\to x_{1}\;\;\;\;\;\;r_{2}^{\prime }:f\;[\{x_{1},x_{2}\}]\to x_{2}\;\;\;\;\;\;r_{3}^{\prime }:f\;[\{x_{3}\}]\to x_{3}\;\;\;\;\;\;\;\;\;\;\;r_{4}^{\prime }:f\;[\{x_{3},x_{4}\}]\to x_{4}\;\;\;\;\;\;\;\;\;\;\;r_{5}^{\prime }:f\;[\{x_{3},x_{4},x_{5}\}]\to x_{5}\;$$

The RAFs of this system (with F={f} ) then correspond bijectively to the RAFs of the original system (under the bijection $r_{i}↔r_{i}^{\prime }$ for each i ).

***Proof of lemma 4.1.*** We establish the following more general result from which lemma 4.1 follows by taking ψ to be the maxRAF operator ( φ ) for a CRS.

Given any function $\psi :2^{Y}\to 2^{Y}$ define an associated derived function $\tilde{\psi }:2^{Y}\to 2^{Y}$ as follows: For X⊆Y , let $F_{\psi }\,\left(X\right)=\left\{U\subseteq X:\psi \,\left(U\right)=U\right\}$ (the ‘fixed sets’ of ψ ) and set:

(A 2)
$$\tilde{\psi }\,\left(X\right)=⋃\left\{U\in F_{\psi }\,\left(X\right):\psi \,\left(U\setminus \left\{u\right\}\right)=\emptyset ,\forall u\in U\right\}.$$

In words, $\tilde{\psi }\,\left(X\right)$ is the set of elements in X that lie in at least a minimal fixed set of ψ .

**Lemma A.1.** *If* ψ *is an interior operator on* $2^{Y}$ *then* $\tilde{\psi }$ *is also an interior operator on* $2^{Y}$ *, and* $\tilde{\psi }\,\left(X\right)\subseteq \psi \,\left(X\right)$ *for all* $X\in 2^{Y}$ *.*

***Proof*** **.** Condition (*I*_1_) holds for $\tilde{\psi }$ since ψ⁢(X)⊆X for all $X\in 2^{Y}$ and so $U\in F_{\psi }\,\left(X\right)$ implies that U⊆X , and by definition $\tilde{\psi }\,\left(X\right)$ is a union of sets in $F_{\psi }\,\left(X\right)$ . Condition (*I*_2_) holds for $\tilde{\psi }$ since if $X\subseteq X^{\prime }$ and $U\in F_{\psi }\,\left(X\right)$ then $U\in F_{\psi }\,\left(X^{\prime }\right)$ . Thus it remains to establish (*I*_3_). We have $\tilde{\psi }\,\left(\tilde{\psi }\,\left(X\right)\right)\subseteq \tilde{\psi }\,\left(X\right)$ by condition (*I*_1_), and so it suffices to establish the reverse containment. Suppose that $U\in F_{\psi }\,\left(X\right)$ . Then since U⊆X and ψ⁢(U)=U , it follows that U=ψ⁢(U)⊆ψ⁢(X) . Thus,

$$\left\{U\in F_{\psi }\,\left(X\right):\psi \,\left(U\setminus \left\{u\right\}\right)=\emptyset ,\forall u\in U\right\}\subseteq \left\{U\in F_{\psi }\,\left(\psi \,\left(X\right)\right):\psi \,\left(U\setminus \left\{u\right\}\right)=\emptyset ,\forall u\in U\right\},$$

and so the union of the sets on the left (i.e. $\tilde{\psi }\left(X\right))$ ) is a subset of the union of the sets on the right (i.e. $\tilde{\psi }\,\left(\tilde{\psi }\,\left(X\right)\right)$ ). Thus, by equation (A 2) (*I*3) holds for $\tilde{\psi }$ , and so $\tilde{\psi }$ is an interior operator on $2^{Y}$ .

The containment $\tilde{\psi }\,\left(X\right)\subseteq \psi \,\left(X\right)$ for all $X\in 2^{Y}$ follows by observing from equation (A 2) that $\tilde{\psi }\,\left(X\right)$ is a union of a sub-collection of the set of subsets of X that are fixed by ψ , and the union of all such ψ –fixed subsets of X is ψ(X) .∎

***Proof of proposition 3.2*** **.** Suppose that $R_{\mathrm{int}}:={⋂}_{i\geq 1}{\hat{R}}_{i}\neq \emptyset$ . Then $R_{\mathrm{int}}$ is an RAF, and every reaction of $R_{\mathrm{int}}$ has a catalyst $U_{i}$ that is not a subset of F , and so $R_{\mathrm{int}}$ is a strictly autocatalytic RAF. On the other hand, if $R^{\prime }$ is a strictly autocatalytic RAF, then since $R^{\prime }\subseteq R$ we have (by induction on i≥1 ) $R^{\prime }\subseteq {\hat{R}}_{i}$ for each i . Thus, since $R_{\mathrm{int}}={\hat{R}}_{k}$ for some value of k we have $R^{\prime }\subseteq R_{\mathrm{int}}$ and so $R_{\mathrm{int}}$ is the unique maximal strictly autocatalytic RAF for Q .∎

***Proof of proposition 4.1.*** The argument just relies on the interior operator properties of φ established in lemma 3.1. If $\varphi \,\left(\varphi \,\left(R\right)\setminus \left\{r_{1},\ldots ,r_{k}\right\}\right)\neq \emptyset$ then $\varphi \,\left(R\right)\setminus \left\{r_{1},\ldots ,r_{k}\right\}$ contains an iRAF, and this iRAF cannot be one of $R_{1},\ldots ,R_{k}$ , since for each i , *r*_*i*_ was chosen from $R_{i}$ yet $r_{i}\notin \varphi \,\left(\varphi \,\left(R\right)\setminus \left\{r_{1},\ldots ,r_{k}\right\}\right)$ by definition. Conversely, if there is an iRAF $R^{\prime }$ of R that is different from $R_{1},\ldots ,R_{k}$ , then selecting *r*_*i*_ in $R_{i}\setminus R^{\prime }$ for each i (this is possible since $R_{i}$ is not a subset of $R^{\prime }$ ), $R^{\prime }$ is an iRAF of $\varphi \,\left(R\right)\setminus \left\{r_{1},\ldots ,r_{k}\right\}$ and so $R^{\prime }=\varphi \,\left(R^{\prime }\right)\subseteq \varphi \,\left(\varphi \,\left(R\right)\setminus \left\{r_{1},\ldots ,r_{k}\right\}\right)$ , thus this set is non-empty. ∎

***Proof of proposition 4.2*** **.** First note that the problem described lies in the complexity class NP, since it can readily be verified (in polynomial time in |𝒬| ) whether or not a given subset of reactions is an iRAF for 𝒬 that contains a given reaction. We reduce an instance of the problem described to the following graph-theoretic problem: Given a finite directed graph G=(V,A) , and a vertex v∈V , determine whether or not there exists a chordless cycle in G that contains v . This problem was shown to be NP-complete in [49] (theorem 1).

Given an arbitrary finite directed graph G=(V,A) , consider the following CRS ${𝒬}_{G}=\left(X,R,\chi ,F\right)$ where F={f} , X=V∪F , $R=\left\{r_{v}:v\in V\right\}$ where *r*_*v*_ is the reaction $r_{v}:f\,\,\left[u_{1},\ldots ,u_{n}\right]\to v$ for each ordered pair $\left(u_{i},v\right)\in A$ . Notice that ${𝒬}_{G}$ is a CRS for which there is a single reactant for each reaction (namely f ) which comprises the food set. Moreover, each reaction has singleton catalysts (i.e. complex catalysis is not involved).

Now any CRS 𝒬=(X,R,χ,F) that has the property that (i) each of its reactions has all its reactants in the food set, and (ii) each catalyst of each reaction is a singleton element in X∖F , then the iRAFs for 𝒬 correspond precisely to the subsets of R that form a chordless cycle of the graph $G_{𝒬}:=\left(R,A\right)$ where $\left(r,r^{\prime }\right)\in A$ if a product of r is a catalyst of $r^{\prime }$ [26, theorem 2.1(ii)]. It follows that the iRAFs for ${𝒬}_{G}$ are precisely the chordless cycles of G , which establishes the claimed reduction.∎

***Proof of proposition 4.3*** **.** Suppose that $R^{\prime }$ is a minimal subset of maxRAF(Q) that is an RAF and produces all of the elements $x_{1},\ldots ,x_{k}$ . Then $R^{\prime }$ is an RAF of ${𝒬}^{+}:={𝒬}^{+\left\{x_{1},x_{2},\ldots ,x_{k}\right\}}$ . Moreover, if $R^{\prime \prime }$ is a strict subset of $R^{\prime }$ that is both n RAF and that produces $x_{1},\ldots ,x_{k}$ , then $R^{\prime \prime }$ is also an RAF of ${𝒬}^{+}$ and so $R^{\prime }$ is not a minimal set with the joint properties of being an RAF and producing $x_{1},\ldots ,x_{k}$ . Thus $R^{\prime }$ is an iRAF of ${𝒬}^{+}$ . Conversely, suppose that $R^{\prime }$ is an iRAF of ${𝒬}^{+}$ . Then $R^{\prime }$ is an RAF of 𝒬 and $R^{\prime }$ must produce all of the elements $x_{1},\ldots ,x_{k}$ (since all reactions in ${𝒬}^{+}$ require each of the elements $x_{1},\ldots ,x_{k}$ to be produced because none of these elements lie in the food set F (by assumption)). Moreover, $R^{\prime }$ is minimal subset of R that is an RAF for 𝒬 and produces $x_{1},\ldots ,x_{k}$ ; for otherwise $R^{\prime }$ contains a strict subset with these properties and so would be an RAF of ${𝒬}^{+}$ contradicting the assumption that $R^{\prime }$ is an iRAF of $Q^{+}$ .∎

***Proof of proposition 4.4.*** We begin with a lemma.

**Lemma A.2.** *Suppose that* $R^{\prime }$ *and* $R^{\prime \prime }⊊R^{\prime }$ *are both RAFs for* 𝒬=(X,R,χ,F) *. Then* $R^{\prime }\setminus R^{\prime \prime }$ *is an RAF for* $\left(X,R,\chi ,F\cup \pi \,\left(R^{\prime \prime }\right)\right)$ *.*

To see why this holds, simply observe that if $o^{\prime }$ is an admissible ordering for $R^{\prime }$ and $o^{-}$ is the induced ordering of $R^{\prime }\setminus R^{\prime \prime }$ then $o^{-}$ is an admissible ordering of $R^{\prime }\setminus R^{\prime \prime }$ for the CRS with expanded food set $\left(X,R,\chi ,F\cup \pi \,\left(R^{\prime \prime }\right)\right)$ .

Returning to the proof of proposition 4.4, observe that (ii) and (iii) are equivalent (by the comments following the last lemma) so it suffices to show that (iii) implies (i), and, conversely, that (i) implies (iii). Suppose first that (iii) holds. Since $R^{\prime }$ equals the (disjoint) union of the sets ${\hat{R}}_{i}$ we can select an admissible ordering of ${\hat{R}}_{i}$ for each i , and extend this to an ordering of the reactions of $R^{\prime }$ in which all reactions from ${\hat{R}}_{i}$ come before those of ${\hat{R}}_{i+1}$ for each i . This ordering is an admissible ordering of $R^{\prime }$ , and each reaction in $R^{\prime }$ is either catalysed by an element of the food set or by the product of another reaction from $R^{\prime }$ . Thus $R^{\prime }$ is an RAF for 𝒬 .

The proof of the converse direction ((i) implies (iii)) follows from lemma A.2, by induction on the length of the sequence $R_{1},R_{2},\ldots$ and the fact that every RAF contains an iRAF. ∎
